# Application of Variable Life Adjusted Displays (VLAD) on Victorian Admitted Episodes Dataset (VAED)

**DOI:** 10.1186/1472-6963-12-278

**Published:** 2012-08-28

**Authors:** Nick Andrianopoulos, Damien Jolley, Sue M Evans, Caroline A Brand, Peter A Cameron

**Affiliations:** 1 Department of Epidemiology & Preventive Medicine, Centre for Research Excellence in Patient Safety, School of Public Health, Preventive Medicine Monash University, Melbourne, Australia

## Abstract

**Background:**

The need to improve patient safety has been identified as a major priority for health reform in developed countries, including Australia. We investigated the implementation and appropriateness of Variable Life Adjusted Displays as a quality control procedure to monitor “in-control” versus “out-of-control” processes in Victorian public hospitals.

**Methods:**

Victorian Admitted Episode Data from Department of Human Services, Victoria for 2004–7 were used. The VLAD is a plot of a cumulative sum of the difference in expected outcome (range 0–1) and observed outcome (0 or 1) for sequential separations. Three outcomes were assessed: in-hospital mortality for acute myocardial infarction, stroke and heart failure. Logistic regression was used to obtain a realistic measure of expected mortality over the period 2004–5, adjusting for covariates and comorbidities, to estimate expected mortality risk for the separations between 2005–7. VLAD were plotted for the years 2005–7, by the 11 hospitals with the highest frequency of separations. Signalling limits for 30%, 50% and 75% risk decrease and risk increase were determined and plotted for each VLAD utilizing risk-adjusted cumulative sum techniques. This is a likelihood-ratio test statistic for signalling. If the VLAD signalled by intersecting with a limit, the limit was reset.

**Results:**

The three logit models displayed reasonable fit to the observed data. There were n = 2999 separations in the AMI model, n = 3598 in the HF model and n = 1922 in the stroke model. The number of separations plotted by VLAD ranged from n = 126 to n = 648. No signals were observed in 64%, 55% and 18% of VLAD for AMI, HF and stroke respectively. For AMI and HF 9% of hospitals signalled at least once for each of 30%, 50% and 75% risk increase, whereas this was 45% for stroke. Hospitals signalling at least once for risk decrease ranged from 18% to 36% across the levels of risk and outcomes. No VLAD signalled for both risk decrease and increase.

**Conclusions:**

VLAD intersecting with limits to signal “out-of-control” states, may be an appropriate technique to help hospitals assess quality control. Preliminary work displays some between hospital differences. Relevant signals can be used to investigate why a system is potentially performing better than or worse than expected. Types and levels of investigation could depend on the type of signalling. Validation work, for example attempting to correlate signals with clinical notes, prior to VLAD distribution needs to be undertaken.

## Background

The need to improve patient safety has been identified as a major priority for health reform in developed countries, including Australia [1]. There are various types of indicators that can be developed and used in the setting of measuring quality and improving outcome [2], however some studies suggest little evidence of reductions in adverse events, despite extensive efforts to improve patient safety [3].

Retrospective application of clinical indicators has highlighted the potential to identify harm in a timely manner, as exemplified by well documented cases of Harold Shipman [4], Bristol [5], and Bundaberg [6]. Such high profile cases alert clinicians and administrators that differences in outcome may emerge and not be noticed. The pyramid model of investigation to find credible cause for high mortality of patients [7] was adopted by Mohammed et al. in their retrospective analysis of routine data to investigate general practitioners associated with high patient mortality flagged through the Shipman enquiry [8].

Variation between organisations can be identified and displayed using a number of different methods. These include Shewart charts, moving average plots and cumulative sum (CUSUM) charts [9], funnel plots [10], resetting sequential probability ratio tests (RSPRT) [11], cumulative risk adjusted mortality (CRAM) charts [12] and VLAD [13]. A crucial element for any analysis of variation is appropriate risk-adjustment for case mix with a view to estimate a realistic probability of outcome [14-16]. Ideally any statistical charting technique would alert to significant variation early.

In-hospital mortality is often reported as a measure of quality of care [17]. It has been widely used internationally because it is relatively easy to measure using existing data and has good face validity i.e. hospitals with higher rates of risk adjusted mortality would be expected to demonstrate poorer quality of care.

We investigated the application and appropriateness of Variable Life Adjusted Displays (VLAD) as a quality control procedure to monitor “in-control” versus “out-of-control” states in Victorian public hospitals. An “out-of-control” state could be suggested by use of statistical process-control methods by identification of excess numbers of infrequent events [18].

A VLAD is a plot of cumulative sum of difference in expected and observed outcome, which also includes upper and lower limits to suggest better than or worse than expected performance, when signalling occurs by intersection of the line and the respective limits. Specifically for this project the scope was to assess whether VLAD for 3 mortality indicators that had been applied to the Queensland Health Admitted Patient Data Collection could be applied to the Victorian Admitted Episode Dataset (VAED).

Our aim for each indicator a priori was to, on 2004–7 VAED data, as outlined by the Queensland Health Quality and Health Program [19], (i) use identical inclusion and exclusion criteria to define denominators and numerator events, (ii) use identical covariates and comorbidities to risk adjust using logistic regression, (iii) determine probability of expected outcome on VAED 2005–7 data, based on 2004–5 logistic regression results and (iv) plot VLAD for sequential separations by hospital on VAED 2005–7 data.

## Methods

The VAED is an administrative dataset maintained by Department of Human Services, Victoria (DHS) and is based upon hospital data compiled by individual public and private hospitals in Victoria, Australia. The dataset contains demographic and clinical information on each discharge, with diagnostic and procedure codes coded in ICD-10-AM [20]. VAED data for all public hospital (n = 45) episodes (separations) were obtained from DHS for the time period July 1st 2004 to June 30^th^ 2007.

For data analysis, identical inclusion and exclusion criteria and risk adjustment for covariates and comorbidities to those used by the Queensland Health Quality and Health Program [19] were used to assess 3 outcomes: in-hospital mortality (as captured by the discharge status field) for (i) acute myocardial infarction (AMI), (ii) stroke and (iii) heart failure (HF). Specific exclusion criteria for each outcome are shown in Table 1. 

**Table 1:** Frequencies of separations and relevant exclusions by indicator

**AMI: 2004-7**		**Number of separations**	**%**
	**Total**	**32,550**	**100**
	LOS <4 days and discharged alive	14,814	46
	LOS >30 days	418	1
	Not admitted through hospital emergency department	8,830	27
	Transfers out	10,616	33
	Age <30 years	67	0
	Age ≥85 years	4,661	14
	**Eligible**	**8,924**	**27**
**Stroke: 2004-7**			
	**Total**	**22,706**	100
	LOS <4 days and discharged alive	6,239	28
	LOS >30 days	1,432	6
	Not admitted through hospital emergency department	4,641	20
	Transfers out	8,071	36
	Age <30 years	311	1
	Age ≥85 years	4,768	18
	Carotid endarterectomies	102	1
	**Eligible**	**5,857**	**26**
**Heart Failure: 2004-7**			
	**Total**	**27,389**	100
	No overnight stay	3,786	14
	LOS >30 days	649	2
	Not admitted through hospital emergency department	6,293	23
	Transfers out	4,510	17
	Age <30 years	141	1
	Age ≥85 years	7,876	29
	**Eligible**	**11,124**	**41**

ICD-10-AM principle diagnosis codes used for each outcome were: (i) I21 and I22 for AMI, (ii) I61, I62, I63 and I64 for stroke and (iii) I50 for heart failure.

Covariate and co-morbidity(ICD codes) used for risk adjustment for each outcome were: (i) age group, gender, dementia(F00-F03; G30-G311), hypotension and shock(I95; R57), renal failure(N17; N18.3; N18.4; N18.5; N18.9; N19; R34), heart failure(I50), dysrhythmias(I46-I49), malignancy(C00-C97), cerebrovascular disease(I60-I69), hypertension(I10-I15) and diabetes(E10-E14) for AMI, (ii) age group, septicaemia(A40-A41), malignancy(C00-C97), heart failure(I50), acute lower respiratory tract infection and influenza(J9-J22) and renal failure(N17; N18.3; N18.4; N18.5; N18.9; N19; R34) for stroke and (iii) age group, septicaemia(A40-A41), malignancy(C00-C97), dementia(F00-F03; G30-G311), hypertension(I10-I15), ischaemic heart disease(I20-I25), dysrhythmias(I46-I49), ), acute lower respiratory tract infection and influenza(J9-J22), ulcer of lower limb or decubitus ulcer(L89; L97), renal failure(N17; N18.3; N18.4; N18.5; N18.9; N19; R34), hypotension and shock(I95; R57) and cerebrovascular disease(I60-I69) for HF. All were used as binary variables, except age group which had multiple categories (most of 5 year range).

We used logistic regression for risk adjustment and we assessed model performance for each indicator using the area under the Receiver Operating Characteristic curve (ROC) and the Hosmer-Lemeshow (H-L) χ^2^ statistic with 8 degrees of freedom for all three years 2004–7 and each year separately. We subsequently calculated expected mortality for 2005–7 VAED separations for each indicator by using the intercept and covariate coefficients for each respective 2004–5 VAED logistic model.

For each of the 11 hospitals with the highest frequency of separations, VLAD were plotted from 2005–7. Cumulative sum of the difference in expected outcome (range 0–1) and observed outcome (0 or 1) was plotted for sequential separations [21]. Signalling limits for 30%, 50% and 75% risk decrease and risk increase were determined and plotted for each VLAD utilizing risk-adjusted CUSUM techniques [22]. This is a log likelihood-ratio test statistic for signalling [23]. If the VLAD signalled by intersecting with a limit, the limit was reset. Appendix 1 outlines the methodology used to plot a VLAD with signalling including the definitions and values used for the variables *ρ* and *h* and associated average run lengths to false alarms (Table 2). We used Stata v10.1 [24] for all analyses and plots.

## Results

Of N = 32550 (AMI), N = 22706 (stroke) and N = 27389 (HF) total separations, N = 8924(27.4%), N = 5857(25.8%) and N = 11124(40.6%) respectively were eligible for analysis following relevant exclusions (Table 1).

Overall observed mortality for eligible separations was 11.9%, 29.8% and 5.5% for AMI, stroke and HF respectively, with similar mortality rates observed in each year (Table 3).

**Table 2:** Observed mortality by indicator and year

	**Frequency of separations**	**% of separations**	**Mortality**	**% of deaths**	**Mortality Risk**	**Risk Ratio**
**AMI**						
2004/5	2999	33.6	338	31.9	11.3%	1.00
2005/6	2977	33.4	371	35.0	12.5%	1.11
2006/7	2948	33.0	350	33.1	11.9%	1.05
**Total**	**8924**	**100**	**1059**	**100**	**11.9%**	**-**
**Stroke**						
2004/5	1922	32.8	540	30.9	28.1%	1.00
2005/6	1966	33.6	609	34.8	31.0%	1.10
2006/7	1969	33.6	599	34.3	30.4%	1.08
**Total**	**5857**	**100**	**1748**	**100**	**29.8%**	**-**
**HF**						
2004/5	3598	32.3	213	34.6	5.9%	1.00
2005/6	3683	33.1	194	31.5	5.3%	0.89
2006/7	3843	34.6	209	33.9	5.4%	0.92
**Total**	**11124**	**100**	**616**	**100**	**5.5%**	**-**

Most covariates in the multivariate logistic models were independent predictors (p < 0.05) of outcome. Females had higher risk of in-hospital mortality following AMI compared to males OR = 1.21(95%CI = 1.04 – 1.40). Age group categories less than the reference category (60–64 years for AMI and 65–69 years for HF and stroke) had lower odds of outcome, while age group categories higher than the reference category had increased odds of outcome. All co-morbidities displayed increased odds of outcome except presence of diabetes for in-hospital AMI, OR = 0.98(95%CI = 0.83 – 1.16) and presence of hypertension for in-hospital AMI and HF, OR = 0.49(95%CI = 0.42 – 0.57) and OR = 0.62(95%CI = 0.51 – 0.74) respectively. There is evidence of year of separation trending towards a significant predictor for in-hospital stroke OR = 1.15(95%CI = 1.00 – 1.33) and OR = 1.13(95%CI = 0.98 – 1.31) for 2005–6 and 2006–7 compared to 2004–5 respectively.

In the 2004–5 models used to calculate subsequent expected risk, there were n = 2999 separations in the AMI model, n = 1922 in the stroke model and n = 3598 in the HF model. Risk adjustment for HF appears to be most appropriate with similarly high ROC 0.83 for 2004–5 and 0.81 for 2004–7 and similarly low non-significant H-L χ^2^ = 8.9, p = 0.35 for 2004–5 and 9.2, p = 0.32 for 2004–7. The risk models for stroke had a low area under ROC (0.68 and 0.67) and whilst the models for AMI had high area under ROC, their H-L statistic was also high (χ^2^ = 13.77, p = 0.09 for 2004–5 and χ^2^ = 45.03, p < 0.0001 for 2004–7).

The numbers of separations plotted for the 33 VLAD ranged from n = 126 to n = 648. Table 4 shows, for each outcome, the total number of signals seen, as well as the percentage of hospitals signalling, in the hospital VLAD, suggesting better than expected performance, at levels of 30%, 50% or 75% risk decrease, and suggesting worse than expected performance at levels of 30%, 50% or 75% and risk increase. No signal was observed in 64%, 55% and 18% of VLAD for AMI, HF and stroke respectively. For AMI and HF 9% of hospitals signalled at least once for each of 30%, 5% and 75% risk increase, whereas this was 45% for stroke. Hospitals signalling at least once for risk decrease ranged from 18% to 36% across the levels of risk and outcomes. No VLAD signalled for both risk decrease and increase.

**Table 3:** Number of VLAD signals by condition and percent of N = 11 hospitals signalling at least once for each %risk change for 2005/7 separations

		**Risk Decrease**			**Risk Increase**		
		**30%**	**50%**	**75%**	**30%**	**50%**	**75%**
**AMI**	Total number of signals	4	5	3	1	1	1
	Hospitals signalling at least once	27%	27%	18%	9%	9%	9%
**Stroke**	Total number of signals	5	5	4	11	11	11
	Hospitals signalling at least once	36%	36%	36%	45%	45%	45%
**HF**	Total number of signals	3	5	5	1	1	1
	Hospitals signalling at least once	18%	36%	36%	9%	9%	9%

**Table 4:** **Average run length (ARL) to false alarm and value of ***h ***for varying values of **ρ_*_

	**Improved performance**			**Worse performance**		
***ρ***	0.70	0.50	0.25	1.30	1.50	1.75
*h*	2.6	3.6	4.9	2.8	3.7	5
ARL	229	682	2447	264	834	3118

*adapted from Coory *et al.*[22]

**Figure 1:**
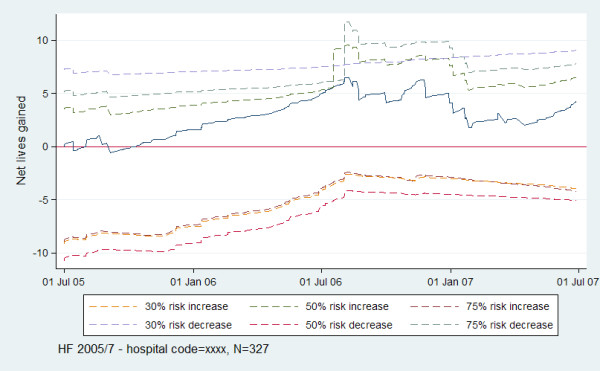
VLAD with upper and lower risk-adjusted CUSUM limits for hospital code = xxxx for HF in-hospital mortality for 2005/7.

**Figure 2:**
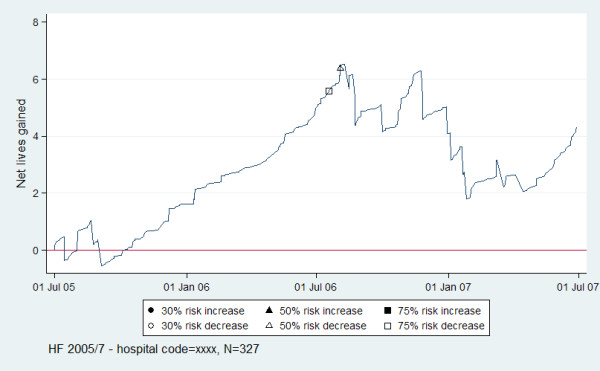
VLAD with upper and lower risk-adjusted CUSUM limit signals for hospital code = xxxx for HF in-hospital mortality for 2005/7.

Figures 1 (dashed lines for limits) and Figure 2 (shapes for signals) show examples of two methods of displaying limits and signalling for VLAD display. The example VLAD signals, suggesting statistically significantly better than expected performance, first at 75% risk decrease and then at 50% risk decrease when compared to the VAED data as a whole, at around July 2006. At the time of the second signal, the hospital had approximately 6 cases of mortality less than expected. The period of the continuous increase in slope eventually resulting in signalling (suggesting the investigation range) was from approximately August 2005 – July 2006.

## Discussion

We have shown that the VAED can be applied in developing an indicator to potentially assess quality control. Intersections between the VLAD curve and the limits to signal “out-of-control” states could be an appropriate technique to help hospitals assess individual variation from an overall average. Relevant signals can be used to investigate why a system is potentially performing better than or worse than expected. Types and levels of investigation could depend on the type of signalling.

We recognise that only the technical aspects of VLAD production have been demonstrated within the scope of this proof of concept. We have shown that we can produce technically correct VLAD charts, and we have identified the levers, parameters and other controls which must be decided and set for implementation.

Many questions remain unanswered, before control chart technology can be implemented across the Victorian public health system. There are the obvious resource questions – how much effort and technology infrastructures (hardware/software) are needed, both for production and distribution of VLAD charts, and for their management within a ‘signalling’ hospital? But there also remain important questions of interpretation and utility.

We recognise that VLAD is likely to be a clinically appealing and potentially useful tool to monitor possible out-of-control conditions in Victorian hospitals. VLAD is a control chart that can be implemented as a measure of quality control in an attempt to differentiate special cause variation from common cause variation in a system [25]. Special cause variation refers to variation as a result of incorrect implementation of an otherwise normal system, whereas common cause variation is expected as part of normal day-to-day variability.

The purpose of implementing VLAD not only includes monitoring for out-of-control states, but also includes reassurance that existing systems are in control. VLAD will be used as a monitoring tool that may initiate an appropriate investigative response. In implementation, it is just as important to investigate why a certain hospital is performing better than expected, so that successful procedures can be implemented elsewhere, than just investigating why another hospital appears to be performing worse than expected.

While VLAD technology has the potential to open up new opportunities for monitoring quality of hospital care in Victoria, we need to be sure that the methods produce results which are interpretable and which do not overwhelm our hospitals with false positive signals. It is essential, therefore that appropriate processes for interpretation, review and feedback are implemented before a potentially damaging “production line” is generated. All results must be reviewed intensively for their quality, accuracy and interpretability before distribution.

Measurement of both observed and expected outcome can be biased. Coding errors can occur in variables that are used to measure both; however case mix adjustment is widely done because adjusted comparisons are generally considered to be less biased than unadjusted comparisons [26]. Observed outcome can be further biased if mortality occurs soon after hospital discharge.

Critical to the validity of VLAD is an appropriate estimation of expected outcome. Overestimating expected outcome may lead to increased frequency of signalling for better than expected performance, while increased frequency of signalling for worse than expected performance may be observed if expected outcome is underestimated.

This study has focused on developing risk models as used in Queensland based on 2004/5 data and applying to VLAD on 2005/7 data. This may not be a suitable long term method of expected risk calculation for data from the VAED. Bootstrapping techniques [27] to discover the best risk model for each indicator may be employed based on the data available, however appropriate clinician input for valid risk adjustment is also important. Should the VAED not contain certain variables that are considered necessary for appropriate risk adjustment, then appropriate merging with or use of other data sources e.g. clinical registries [28] may be considered.

Expected risk may change over time. It may be appropriate to implement a rolling logistic regression risk model. This may simply involve using the previous 12, 24 or 36 months (whole units of year needed to account for seasonal variation) of data for the purpose of determining expected risk with the same adjustment variables, or it may involve revisiting model development and adjusting variables in the model as necessary.

For each indicator, fine tuning of *ρ* and *h* is needed to minimize false positives and attempt to avoid false negatives. Assumptions that a system is in control at a start of a VLAD may need to be made. More rigorous limits e.g. *h/2* could be set initially to observe signalling. Review of subsequent VLAD could occur in two ways. (i) as a cumulative addition to the previous VLAD and (ii) as a new VLAD starting from zero in its own right, may help to address methodological questions about performance of the curves and resetting. Assessment of the relative strengths and weaknesses of these after several cycles would be helpful.

Three sets of signals each for risk increase and risk decrease have been implemented, with the view to implementing a tiered response to signals. Recommended initial responses to signals at each level need to be established and validated. Queensland Health’s current flag levels for various indicator groupings and responses to flag level signalling are outlined in their VLAD implementation standard [29]. Their use recently led to refinement of the laparoscopic cholecystectomy complications of surgery indicator definition [30].

Early signalling to suggest significant variation is a desirable goal for VLAD, however simulation studies suggest ability to signal early may be mild and may be strictly correlated with the institution volume of activity [31]. Hence the authors conclude it may be preferable to use an integration of VLAD and another tool e.g. CUSUM charts. Furthermore, Scott et al. conclude that appropriate patient selection may be more important than choice of dataset or risk-prediction model when statistical process-control methods are used to flag unfavourable mortality trends suggestive of sub-optimal hospital care [18].

This study is further limited in that we have assessed VLAD using only one dataset and within that only hospitals with the highest volume of admissions. There has also been no attempt to check whether the signals actually represent significant variation in practice; however the methodology does display feasibility and a way forward.

### Future directions

Assessment of the implications of differences in risk adjustment (both in variables included and temporal changes) on VLAD and their signals. Possibility of use of linked datasets for risk adjustment. Clinical validation and utility of VLAD distribution needs to be undertaken and ultimately an assessment of whether responses actually lead to a change in practice and better quality of care.

## Conclusions

VLAD intersecting with limits to signal “out-of-control” states, may be an appropriate technique to help hospitals assess quality control. They are a relatively straightforward visual representation which may enhance the likelihood of engaging clinicians and administrators. Preliminary work displays some between hospital differences. Relevant signals can be used to investigate why a system is potentially performing better than or worse than expected. Types and levels of investigation could depend on the type of signalling. Validation work attempting to correlate signals with clinical notes, as well as further risk adjustment work, prior to VLAD distribution needs to be undertaken.

## Appendix 1: VLAD Methodology

Using the logistic regression to predict a risk coefficient (*p*) for a patient for a set of *n* covariates:

(1) $$\text{logit}\left(p\right)=\beta_{0}+\sum_{i=1}^{n}\beta_{i}x_{i}$$

Expected outcome (*E*) can be calculated as:

(2) $$E=\frac{e^{p}}{1+e^{p}}$$

And Observed outcome (*O*) is *O* = 0 if the patient survives, *O* = 1 if the patient dies.

A VLAD is a curve that plots cumulative (*E*) – (*O*) events:

(3) $$V_{n}=\sum_{i=1}^{n}E_{i}-\sum_{i=1}^{n}O_{i}$$

VLAD limits can be calculated from:

For the lower limit the CUSUM of the *n*th observation (*C*_n_) with the corresponding weight *W*_n_ is given by:

C_0_ = 0 and

(4) $$C_{n}=\max\left\{C_{n-1}+W_{n},0\right\}$$

Where

(5) $$W_{n}=O_{n}\log\rho -\log\left(1+\left(\rho -1\right)E_{n}\right)$$

where *ρ* is the ratio of risk under the alternative and null hypotheses.

The lower VLAD limit (*L*_n_) can then be calculated by the expression:

(6) $$L_{n}=V_{n}+\left(C_{n}-h\right)/\log\rho$$

where *h* is a control limit signifying when the CUSUM signals.

Should *V*_n_ intersect with *L*_n_, the limit is reset to *Z*_n_ by:

(7) $$Z_{n}=L_{n}+h/\log\rho$$

For the upper limit, the CUSUM is modified to be:

(8) $$C_{n}=\min\left\{C_{n-1}-W_{n},0\right\}$$

and the limit is modified to be:

(9) $$L_{n}=V_{n}-\left(C_{n}+h\right)/\log\rho$$

## Competing interests

The author(s) declare that they have no competing interests.

## Authors’ contributions

NA: Statistical methodology and analyses, interpretation of results, drafting of manuscript. DJ: Statistical methodology, interpretation of results, drafting of manuscript. SME, CAB, PAC: Interpretation of results, drafting of manuscript. All authors read and approved the final manuscript.

## Pre-publication history

The pre-publication history for this paper can be accessed here:

http://www.biomedcentral.com/1472-6963/12/278/prepub
